# Protective Effect of Methane-Rich Saline on Acetic Acid-Induced Ulcerative Colitis via Blocking the TLR4/NF-*κ*B/MAPK Pathway and Promoting IL-10/JAK1/STAT3-Mediated Anti-inflammatory Response

**DOI:** 10.1155/2019/7850324

**Published:** 2019-04-28

**Authors:** Guanghui Wang, Bing Xu, Feiyu Shi, Mengfan Du, Yaguang Li, Tianyu Yu, Lihong Chen

**Affiliations:** ^1^Department of General Surgery, The First Affiliated Hospital of Xi'an Jiaotong University, Xi'an, 710061 Shaanxi Province, China; ^2^Department of Immunology and Laboratory, Shaanxi University of Chinese Medicine, Xianyang, 712046 Shaanxi Province, China; ^3^International Medical Center, The First Affiliated Hospital of Xi'an Jiaotong University, Xi'an, 710061 Shaanxi Province, China

## Abstract

Ulcerative colitis (UC) is an inflammation-related disease involved in uncontrolled inflammation and oxidative stress and is characterized by high recurrence and relapse risk. As a rising star in gas medicine, methane owns the properties of anti-inflammation, antioxidation, and antiapoptosis. Based on the possible mechanism, we aimed to investigate the effect of methane on UC. Methane-rich saline (MRS) was introduced here, and UC was induced by acetic acid. All the C57BL/6 mice were allocated into groups as follows: control group, colitis model group, colitis treated with salazosulfapyridine (SASP) group, and colitis treated with MRS (1 or 10 ml/kg) groups. Tissue damage, the degree of inflammation, oxidative stress, and apoptosis were evaluated in the study, as well as the TLR4/NF-*κ*B/MAPK and IL-10/JAK1/STAT3 signaling pathways for further exploration of the potential mechanism. The results showed that MRS (1) alleviated tissue damage caused by acetic acid, (2) controlled acetic acid-induced inflammation, (3) inhibited acetic acid-caused oxidative stress, (4) reduced colonic cell apoptosis due to acetic acid, (5) suppressed the TLR-4/NF-*κ*B/MAPK signaling pathway, and (6) activated IL-10/JAK1/STAT3 anti-inflammatory response to improve the injury induced by acetic acid. We conclude that MRS has a protective effect on acetic acid-induced ulcerative colitis in mice via blocking the TLR4/NF-*κ*B/MAPK signaling pathway and promoting the IL-10/JAK1/STAT3-mediated anti-inflammatory response.

## 1. Introduction

Ulcerative colitis (UC), which belongs to the chronic nonspecific inflammatory bowel disease (IBD) family related to immunization, is inflammation in the mucosa and subserosa of the colon and rectum. The main manifestations of UC are diarrhea, mucopurulent bloody stools, abdominal pain, recurrence, and relapse. Additionally, severe UC will result in complications such as a toxic megacolon and an increased risk of colorectal cancer. It was indicated that the prevalence and incidence of UC have been increasing gradually in recent years [1, 2]. UC has been calling for increasing concern because of its damage to individual lives and work capacity. However, the etiology of UC remains unclear, and multiple factors are considered, among which environmental factor, genetic factor, and microbial factor have gained wide acknowledgment.

Inflammation and oxidative stress are thought to play key roles in the pathophysiological process of UC. Stimulation from the outside or inside induced the activation of inflammation, leading to the oxidative stress and aggravation of inflammatory cells [3]. With proinflammatory peculiarity, the products of oxidative stress contribute to destroying cell structure by lipid peroxidation, eventually causing cellapoptosis and necrosis [4]. Dead cells become new stimulation, and a vicious circle is started. Pivotal proinflammatory cytokines such as tumor necrosis factor-*α* (TNF-*α*) and interleukin-6 (IL-6) have been applied to target UC [5]. However, an increased risk of infection and malignancies caused by immune suppression cannot be overlooked. Furthermore, it has been well demonstrated that nuclear factor *κ*B (NF-*κ*B) and mitogen-activated protein kinase (MAPK) pathways are active in the inflammatory response by assisting the function of inflammatory cytokines. Targeting the TLR4/NF-*κ*B/MAPK signaling pathway might be an alternative method to treat inflammatory diseases [6]. Meanwhile, IL-10, one of the best-studied anti-inflammatory cytokines, also contributed to the progress of colitis. The IL-10/JAK1/STAT3-mediated anti-inflammatory response is an essential negative regulator that controls the degree and duration of inflammation [7]. Efforts have been made to explore potential therapy for UC patients, and, herein, we would like to focus on a novel method, methane medicine.

As the simplest alkane, methane (CH_4_) has been thought to be useless to humans for decades until researchers have recently revealed its physiological actions. Previous reports have offered evidence of methane to improve ischemia reperfusion injury, sepsis, acute lung injury, and autoimmune hepatitis, and a positive outcome was even obtained in diabetic retinopathy [8–13]. Researchers found that the anti-inflammation, antiapoptosis, and antioxidation properties of methane make it a protector in those diseases [14]. Generally, methane is used by inhalation or injection of methane-rich saline (MRS). Considering the risk of methane gas explosion, some researchers prefer the latter route. Nevertheless, little attention has been paid to the biological function of methane in UC. Thus, we aimed to explore the curative effect of MRS on UC in this study.

## 2. Materials and Methods

### 2.1. Animals and MRS Preparation

Four- to five-week-old wild-type male C57BL/6 mice (21-25 g) were purchased from the Animal Feeding Center of Xi'an Jiaotong University Health Science Center. Right before the experiment, all the animals were kept in controlled circumstances where five mice shared a room at most with a 12 h light/dark cycle, stationary temperature of 21 ± 2°C, and 50% humidity. Providing with standard food and tap water, the animal care principle was to minimize the discomfort to mice. All animal experiments obeyed the guidelines of the China Council on Animal Care and Use. All the procedures of animal feeding and handling in the study were reviewed, approved, and supervised by the Institutional Animal Care and Use Committee of the Ethics Committee of Xi'an Jiaotong University Health Science Center, China.

Methane was dissolved in sealed normal saline and underwent high pressure (0.4 MPa) for 8 hours to produce MRS. Prepared MRS was reserved using an aluminum bag at 4°C and sterilized by *γ*-radiation one day before utilization. The concentration of MRS was detected using gas chromatography, and the concentration of methane in the MRS was 1.2-1.5 mmol/l.

### 2.2. Experimental Design

The mice were separated into 5 groups at random with 6 animals in each group. Groups 1 (control group) and 2 (AA group) were pretreated with 0.9% saline at a dose of 50 ml/kg. In group 3 (AA+SASP group), the mice were given SASP at a dose of 500 mg/kg. Groups 4 (AA+MRS 1 group) and 5 (AA+MRS 10) received pretreatment with MRS at 1 or 10 ml/kg, respectively. All the drugs were administered by gastric gavage once a day. On the eighth day, the mice from groups 2 to 5 were subjected to colitis induction, while group 1 received a similar procedure with an equal volume of saline. The animals were sacrificed by isoflurane (5%) inhalation 24 h later. Subsequently, blood and tissue samples were collected.

### 2.3. Colitis Induction

The classical method was used here to induce colitis [15]. Mice were anesthetized by ether after fasting for 24 h. Next, a flexible catheter (3.5 F) was inserted into the colon with a depth of approximately 3 cm away from the anus. Acetic acid solution was responsible for inducing UC by being injected into the lumen of the colon intrarectally with a volume of 1 ml and dose of 5%. Next, the mice were kept in a supine Trendelenburg position for 30 s to prevent the leakage of the instilled solution. Mice from group 1 obtained the same treatment using 0.9% saline.

### 2.4. Assessment of Tissue Damage Macroscopically

The disease activity index (DAI) was calculated and consisted of three parameters, namely, weight loss, consistency of stool, and gross rectal bleeding. The assessment criteria were [16] weight loss (0, none; 1, reduced 1-5%; 2, reduced 5-10%; 3, reduced 11-15%; and 4, reduced over 15%), stool consistency (0, normal; 2, loose stool; and 4, watery diarrhea), and rectal bleeding (0, normal; 2, mild bleeding; and 4, severe bleeding). Herein, body weight on the particular day was compared with that on the initial day. The sum of all the grades was evaluated for each animal daily. Additionally, the ulcer area was recorded, and the ulcer index (UI) for each colon sample was calculated as follows: UI = [ulcerated area/total colon area] × 100 [17]. The length and weight of the colon between the ileocecal junction and rectum were measured, and the length/weight ratio was evaluated, as well as the spleen weight.

### 2.5. Assessment of Microscopic Damage

To evaluate the histological change, colon tissue was collected and fixed in 10% formalin solution. Next, the samples were embedded in paraffin, and consecutive 5 *μ*m thick sections were then stained with hematoxylin and eosin. The assessment of microscopic damage referred to the grading system as described previously [18]: (1) loss of mucosal structure (0-3), (2) infiltration of leukocyte (0-3), (3) muscle incrassation (0-3), (4) formation of crypt abscess (0-3), and (5) loss of goblet cells (0-3). The maximum score was 15. Each slide was examined under a microscope by investigators who were blind to the treatment.

### 2.6. Biochemical Assay

#### 2.6.1. Inflammation Parameter

Blood samples were collected to measure the levels of TNF-*α* and IL-6 using enzyme-linked immunosorbent assay (ELISA) kits (Lianke, Hangzhou, China) according to the manufacturer's instructions.

Total RNA was isolated from colon specimens using the RNAfast200 kit (Fastagen Biotech, Shanghai, China). Reverse transcription was performed using the PrimeScript RT reagent kit (TaKaRa Biotechnology, Dalian, China). mRNA expression was assayed in triplicate and normalized to 18S mRNA expression. The relative levels were calculated using the comparative Ct method (ΔΔCt method). The primers used in the study were TNF-*α* (forward 5′-AAG CCT GTA GCC CAC GTC GTA-3′ and reverse 5′-AGG TAC AAC CCA TCG GCT GG-3′), IL-6 (forward 5′-TCC ATC CAG TTG CCT TCT TG-3′ and reverse 5′-TTC CAC GAT TTC CCA GAG AAC-3′), IL-1*β* (forward 5′-GGA GAC TTC ACA GAG GAT AC-3′ and reverse 5′-CCA GTT TGG TAG CAT CCA TC-3′), IL-10 (forward 5′-GCT CTT ACT GAC TGG CAT GAG-3′ and reverse 5′-CGC AGC TCT AGG AGC ATG TG-3′), and 18S (forward 5′-AAA CGG CTA CCA CAT CCA AG-3′ and reverse 5′-CCT CCA ATG GAT CCT CGT TA-3′).

#### 2.6.2. Oxidative Stress Parameter

The colon sections were collected and homogenized. After centrifugation at 5,000 rpm at -4°C for 30 min, the supernatant was separated to investigate common oxidative stress markers in it including malondialdehyde (MDA), myeloperoxidase (MPO), superoxide dismutase (SOD), and glutathione transferase (GSH) via activity assay kits (Nanjing Jiancheng Bioengineering Institute, Nanjing, China) following the manufacturer's protocols.

### 2.7. Evaluation of Apoptosis

The terminal deoxynucleotidyl transferase-mediated nick end labeling (TUNEL) apoptosis assay was performed on colon slides (4 *μ*m thickness) that were embedded by paraffin. Blue appeared when 4′,6-diamidino-2-phenylindole (DAPI) marked the nucleus, and green stood for apoptotic cells. The results were observed by a fluorescence microscope with an emission wavelength of 530 nm and an excitation wavelength of 480 nm, after which ImageJ2x software was used to determine the fluorescence intensity.

### 2.8. Western Blot Analysis

Colon samples were harvested and homogenized in radioimmunoprecipitation assay (RIPA) lysis buffer. The BCA Protein Assay was performed to detect the protein content. Protein samples were separated by 10% sodium dodecyl sulfate polyacrylamide gel electrophoresis (SDS-PAGE) and were transferred onto polyvinylidene fluoride (PVDF) membranes, where they were blocked with 10% skim milk for 1 h and subsequently stained with specific primary antibodies of toll-like receptor 4 (TLR4), myeloid differentiation primary response protein 88 (MyD88), p38 and phosphorylated p38 (p-p38), extracellular signal-regulated kinase (ERK) and phosphorylated ERK (p-ERK), c-Jun N-terminal kinase (JNK) and phosphorylated JNK (p-JNK), nuclear factor *κ*B p65 subunit (NF-*κ*B p65) and phosphorylated NF-*κ*B p65 (p-NF-*κ*B p65), IL-10, Janus kinase 1 (JAK1) and phosphorylated JAK1, and signal transduction and transcription activator 3 and phosphorylated STAT3 (Proteintech Group Inc., China; Beijing Biosynthesis Biotechnology Co. Ltd., China) overnight at 4°C. Next, the membranes were washed three times with PBS. After staining with horseradish peroxidase- (HRP-) conjugated secondary antibodies, the blots were washed again and visualized by chemiluminescence (ECL). The statistical data of the protein levels were generated using ImageJ software with normalization to the level of the *β*-actin control.

### 2.9. Statistical Analysis

All data were recorded as mean ± standard deviation. Statistical analysis was executed by SPSS 18.0 statistical software. Significant differences among groups were confirmed with one-way ANOVA, followed by Fisher's LSD test. *P* < 0.05 was considered statistically significant.

## 3. Results

### 3.1. Effect of MRS on Tissue Damage of Acetic Acid-Induced UC

#### 3.1.1. Spleen Weight

**Figure 1:**
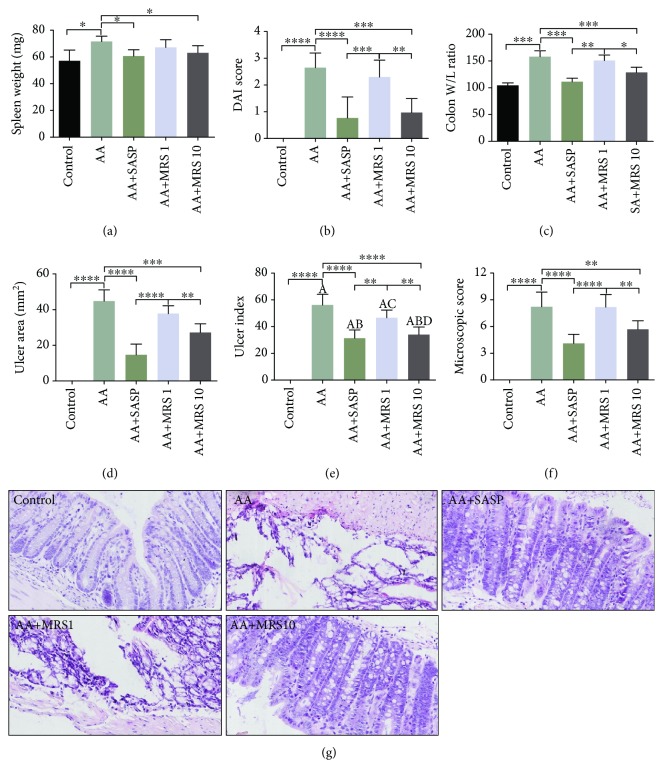
Treatment with MRS mitigated acetic acid-induced tissue damage. Mice were administered with MRS at a dose of 1 or 10 ml/kg by gastric gavage for a week before colitis establishment. SASP was used as a positive control at a dose of 500 mg/kg. Colitis was induced by acetic acid solution (*v*/*v*) injection into the lumen of the colon intrarectally with a volume of 1 ml and dose of 5% except for the sham group. The (a) spleen weight, (b) disease activity index (DAI), (c) colon weight/length ratio, (d) ulcer area, and (e) ulcer index were measured. Additionally, colon tissues were collected to evaluate the damage microscopically by (g) H&E staining (200x) and (f) microscopic scores were calculated. Data were expressed as means ± SD. ^∗^*P* < 0.05, ^∗∗^*P* < 0.01, ^∗∗∗^*P* < 0.001, and ^∗∗∗∗^*P* < 0.0001.

As one of the common manifestations of acetic acid-induced UC, enlargement of the spleen was evaluated by the weight. We found that the spleen weight of mice with colitis showed an increase compared with that of the control group, while SASP and MRS significantly reduced the spleen weight of mice (Figure 1(a)).

#### 3.1.2. Disease Activity Index (DAI)

After the assessment of weight loss, consistency of stool, and gross rectal bleeding in each animal, we obtained a DAI score to judge the symptom severity of UC (Figure 1(b)). We found that the DAI score of group AA was significantly higher than that of any other group, showing that SASP or MRS treatment could relieve the symptoms of UC. Significant difference was shown between the variant dosages of MRS, and mice showed a better DAI score under MRS treatment at 10 ml/kg.

#### 3.1.3. Colon Weight/Length Ratio

The colon weight/length ratio was calculated to assess the condition of colonic mucosal injury. The increased weight/length ratio appeared with a significant difference in animals from group AA compared with those from the naive group. Treatment with SASP and MRS-1 or MRS-10 showed a notable decrease in the colon weight/length ratio as shown in Figure 1(c). However, MRS at a dose of 10 ml/kg showed a significantly lower weight/length ratio than that of 1 ml/kg.

#### 3.1.4. Ulcer Area and Ulcer Index

The ulcer area and ulcer index were calculated to evaluate the severity of ulceration in animals (Figures 1(d) and 1(e)). Mice treated with SASP showed smaller ulcer sites than did untreated colitis mice, as well as smaller ulcer indexes. Although a significant decrease in the ulcer area was shown with either 1 or 10 ml/kg of MRS, only the latter dose reduced the ulcer index with significant difference.

#### 3.1.5. Effect of MRS on Histology Damage of Acetic Acid-Induced UC

H&E staining was utilized here to assess histology changes in animals. We observed colitis under the microscope with cellular infiltration, epithelium destruction, hyperemia, and necrosis compared with normal mice as shown in Figure 1(g). Treatment with SASP and MRS at 1 or 10 ml/kg alleviated the inflammatory microscopic characteristics described above. Similarly, after assessing the microscopic score (Figure 1(f)), we found that the higher scores, which were shown in the AA group compared with those in the control group, were reduced markedly when colitis mice were treated with SASP and MRS (10 ml/kg), indicating that 10 ml/kg of MRS was more effective than the lower dose.

### 3.2. Effect of MRS on the Levels of Inflammatory Cytokines

**Figure 2:**
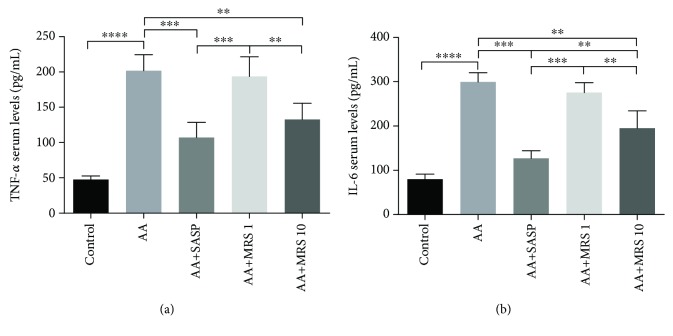
Treatment with MRS reduced the serum levels of inflammatory cytokines in acetic acid-induced UC. Mice were administered with MRS at a dose of 1 or 10 ml/kg by gastric gavage for a week before colitis establishment. SASP was used as a positive control at a dose of 500 mg/kg. Colitis was induced by acetic acid solution (*v*/*v*) injection into the lumen of the colon intrarectally with a volume of 1 ml and dose of 5% except for the sham group. Blood samples were collected to determine the levels of (a) TNF-*α* and (b) IL-6 in the serum. Data were expressed as means ± SD. ^∗∗^*P* < 0.01, ^∗∗∗^*P* < 0.001, and ^∗∗∗∗^*P* < 0.0001.

**Figure 3:**
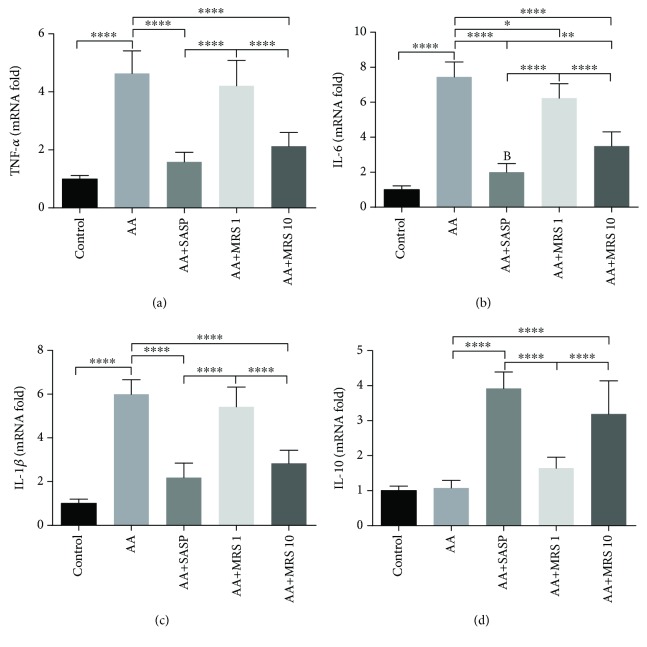
Treatment with MRS inhibited the expression of inflammatory cytokines at the mRNA level in acetic acid-induced UC. Mice were administered with MRS at a dose of 1 or 10 ml/kg by gastric gavage for a week before colitis establishment. SASP was used as a positive control at a dose of 500 mg/kg. Colitis was induced by acetic acid solution (*v*/*v*) injection into the lumen of the colon intrarectally with a volume of 1 ml and dose of 5% except for the sham group. Total RNA was collected from colon specimens, and the expression levels of (a) TNF-*α*, (b) IL-6, (c) IL-1*β*, and (d) IL-10 were tested. Data were expressed as means ± SD. ^∗^*P* < 0.05, ^∗∗^*P* < 0.01, and ^∗∗∗∗^*P* < 0.0001.

TNF-*α* and IL-6 serum levels were elevated significantly due to the stimulation of acetic acid compared with those in the sham group (Figure 2). Treatment with MRS (10 ml/kg), as well as SASP treatment, caused significant decreased levels of such proinflammatory cytokines compared with that in the colitis control group. Further investigation of the cytokine status at the mRNA level produced consistent results that MRS (10 ml/kg) reduced the mRNA level of TNF-*α* and IL-6 markedly (Figures 3(a) and 3(b)). Furthermore, the IL-1*β* mRNA fold change was recorded, showing an obvious increase caused by acetic acid as shown in Figure 3(c), while the level decreased with SASP and MRS treatment at 10 ml/kg. Additionally, as an anti-inflammatory cytokine, IL-10 was found to be decreased notably on the mRNA level when acetic acid is induced (Figure 3(d)). When colitis mice were treated with SASP and MRS (10 ml/kg), the anti-inflammation status was improved by increasing the mRNA level of IL-10 compared with that in the AA group.

### 3.3. Effect of MRS on the Level of Oxidative Stress

**Figure 4:**
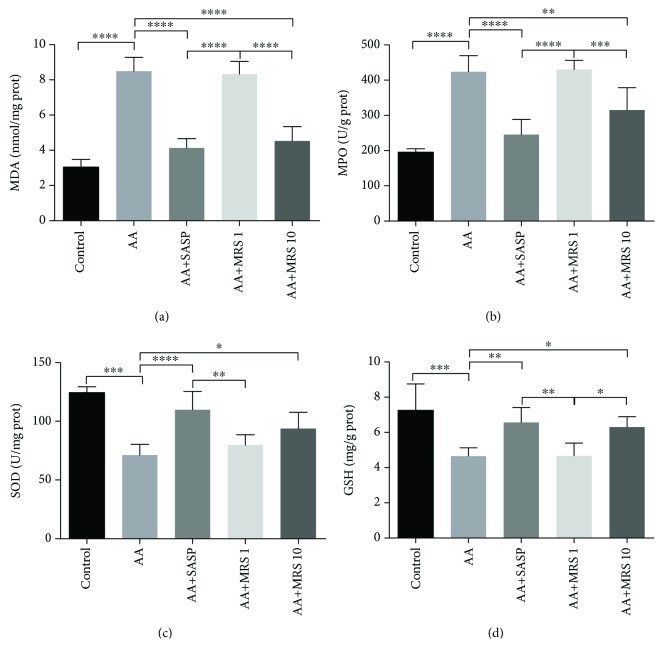
Treatment with MRS suppressed oxidative stress induced by acetic acid. Mice were administered with MRS at a dose of 1 or 10 ml/kg by gastric gavage for a week before colitis establishment. SASP was used as a positive control at a dose of 500 mg/kg. Colitis was induced by acetic acid solution (*v*/*v*) injection into the lumen of the colon intrarectally with a volume of 1 ml and dose of 5% except for the sham group. Colon sections were isolated to investigate the markers of oxidative stress including (a) MDA, (b) MPO, (c) SOD, and (d) GSH. Data were expressed as means ± SD. ^∗^*P* < 0.05, ^∗∗^*P* < 0.01, ^∗∗∗^*P* < 0.001, and ^∗∗∗∗^*P* < 0.0001.

The MDA content revealed the level of lipid peroxidation caused by acetic acid. An elevated MDA content appeared in the AA group compared with that in the control group, which was reduced notably by treatments with SASP and MRS as shown in Figure 4(a). MPO activity was also explored to assess the condition of neutrophil infiltration.  Figure 4(b) shows that the MPO activity of colitis animals was markedly higher than that of the naive group. SASP and MRS treatment all affected the inhibition of MPO activity. Additionally, the activities of endogenous antioxidants SOD and GSH were detected and found to be decreased in the AA group compared with those in the control group (Figure 4(c) and 4(d)). Additionally, SASP and MRS (10 ml/kg) improved the inhibition activities of SOD and GSH with a significant difference.

### 3.4. Effect of MRS on Apoptosis of Acetic Acid-Induced UC

**Figure 5:**
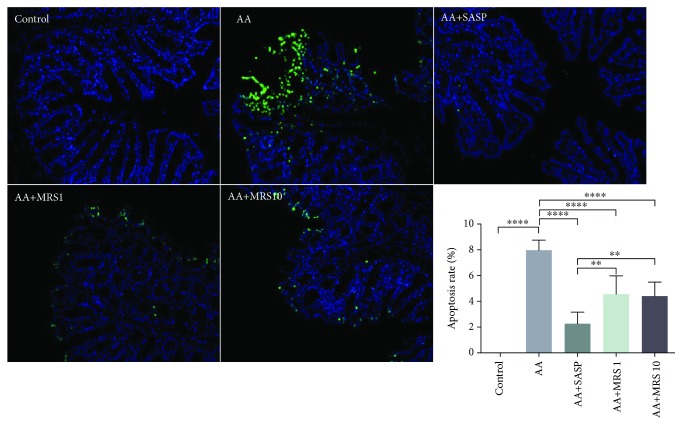
Treatment with MRS improved colonic cell apoptosis caused by acetic acid. Mice were administered with MRS at a dose of 1 or 10 ml/kg by gastric gavage for a week before colitis establishment. SASP was used as a positive control at a dose of 500 mg/kg. Colitis was induced by acetic acid solution (*v*/*v*) injection into the lumen of the colon intrarectally with a volume of 1 ml and dose of 5% except for the sham group. The collected colon slides were subjected to TUNEL staining to assess cell apoptosis, and green spots represented apoptotic cells (400x). And the apoptosis rate was calculated. Data were expressed as means ± SD. ^∗^*P* < 0.05, ^∗∗^*P* < 0.01, ^∗∗∗^*P* < 0.001, and ^∗∗∗∗^*P* < 0.0001.

We explored the effect of MRS treatment on colonic cell apoptosis in acetic acid-induced UC through TUNEL staining. The AA group exhibited a stronger positive fluorescence signal than did the naïve group as shown in Figure 5. Additionally, the samples from the groups treated with SASP or MRS showed fewer positively stained cells. Quantitative measurement was taken, and the result revealed that the apoptotic rate in the colon was significantly reduced under treatment with SASP or MRS.

### 3.5. Effect of MRS on the TLR4/NF-*κ*B/MAPK Signaling Pathway

**Figure 6:**
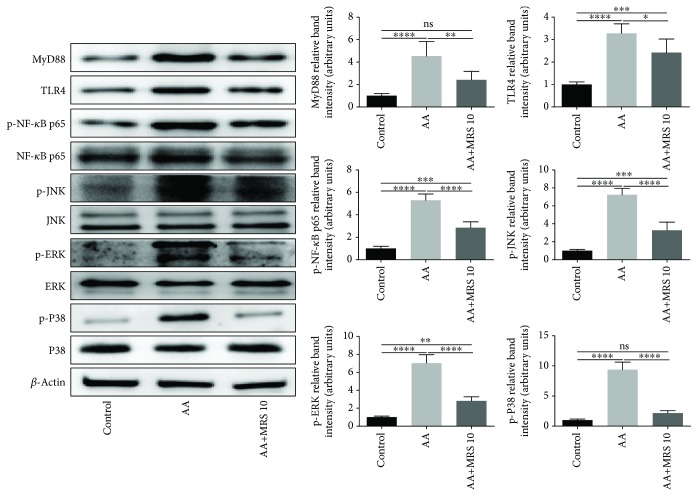
Treatment with MRS downregulated the inflammation-related TLR4/NF-*κ*B/MAPK signaling pathway. Mice were administered with MRS at a dose of 10 ml/kg by gastric gavage for a week before colitis establishment. SASP was used as a positive control at a dose of 500 mg/kg. Colitis was induced by acetic acid solution (*v*/*v*) injection into the lumen of the colon intrarectally with a volume of 1 ml and dose of 5% except for the sham group. Colon tissues were collected, and the protein levels of major pathway components, including TLR4, MyD88, ERK, p-JNK, p38, and NF-*κ*B p65, were analyzed by Western blotting. Data were expressed as means ± SD. ^∗^*P* < 0.05, ^∗∗^*P* < 0.01, ^∗∗∗^*P* < 0.001, and ^∗∗∗∗^*P* < 0.0001.

To elucidate the underlying mechanism of MRS in acetic acid-induced UC, Western blotting was performed to explore the TLR4/NF-*κ*B/MAPKs signaling pathway that was in charge of the inflammatory reaction. As shown in Figure 6, acetic acid increased the expression level of TLR4 and MyD88 in colonic tissues compared with that in the control group, which was markedly reversed by MRS (10 ml/kg). Consistently, we found that the expression levels of p-NF-*κ*B p65, p-JNK, p-ERK, and p-P38 were enhanced with acetic acid but were all significantly suppressed under treatment with MRS (10 ml/kg).

### 3.6. Effect of MRS on IL-10/JAK1/STAT3-Mediated Anti-inflammatory Response

**Figure 7:**
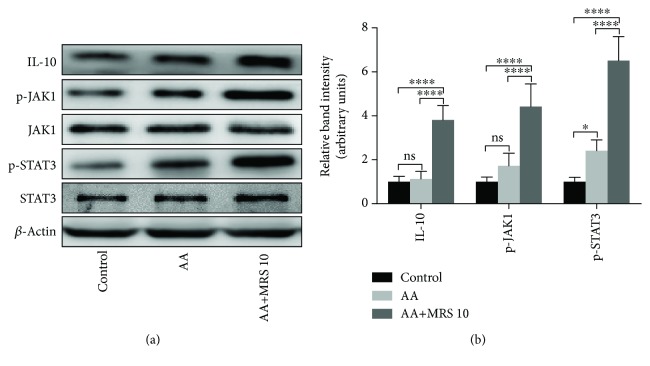
Treatment with MRS activated the anti-inflammatory response-related IL-10/JAK1/STAT3 signaling pathway. Mice were administered with MRS at a dose of 10 ml/kg by gastric gavage for a week before colitis establishment. SASP was used as a positive control at a dose of 500 mg/kg. Colitis was induced by acetic acid solution (*v*/*v*) injection into the lumen of the colon intrarectally with a volume of 1 ml and dose of 5% except for the sham group. Colon tissues were collected, and the protein levels of IL-10, p-JAK1, JAK1, p-STAT3, and STAT3 were analyzed by Western blotting. Data were expressed as means ± SD. ^∗^*P* < 0.05 and ^∗∗∗∗^*P* < 0.0001.

On the basis of the effect of MRS on the transcriptional level of IL-10 which is a vital anti-inflammatory cytokine, we detect the protein expression of IL-10 and its downstream pathway molecules JAK1 and STAT3. The results showed that acetic acid did not significantly affect the expression levels of IL-10 and p-JAK1 but could increase the p-STAT3 expression that might be the initiation of endogenous protection. However, MRS (10 ml/kg) treatment could significantly increase the expression levels of IL-10/p-JAK1/p-STAT3 (Figure 7).

## 4. Discussion

In our study, we investigated the effect of methane-rich saline (MRS) on colitis caused by acetic acid. The results were as follows: (1) MRS alleviated tissue damage in acetic acid-induced UC, (2) MRS controlled inflammation in acetic acid-induced UC, (3) MRS inhibited oxidative stress in acetic acid-induced colon, (4) MRS reduced cell apoptosis in acetic acid-induced UC, (5) MRS protected acetic acid-induced colitis through the TLR-4/NF-*κ*B/MAPKs signaling pathway, and (6) MRS improved anti-inflammatory response by promoting the IL-10/JAK1/STAT3 signaling pathway. Thus, for the first time, the prospect of MRS as a therapeutic method for ulcerative colitis is discussed.

Ulcerative colitis was established by acetic acid in the study and is a classical method that is widely used to imitate the pathophysiologic process of UC with high operability. When assessing the effect of MRS on colitis manifestation, we found a decrease in the spleen weight, DAI score, colonic weight/length ratio, ulcer area, and index. Together with positive microscopic results, MRS showed its ability to alleviate the tissue damage caused by acetic acid.

It was proven that colitis is closely related to inflammation [19]. The caspase cascade of inflammation is elicited during colitis and is characterized by the recruitment of inflammatory cells and release of cytokines to fight against stimulation and damage to the tissue simultaneously. Herein, TNF-*α* plays an essential role in triggering the production of a series of chemical mediators, leading to stronger inflammation [20]. Additionally, IL-6 is a key molecule contributing to neutrophil infiltration and cell apoptosis in colitis [21]. TNF-*α* and IL-6 serum levels were elevated in the colitis group as expected but were reduced by MRS. At the molecular to mRNA levels, the reduction of the TNF-*α* and IL-6 mRNA levels under MRS treatment suggested the anti-inflammatory effect of MRS again. The mRNA fold of IL-1*β* was also detected because of its important role in proinflammatory cytokines in colitis. Additionally, the mRNA fold of IL-1*β* was decreased with MRS. Furthermore, the expression level of IL-10, a well-known anti-inflammatory cytokine, was upregulated by MRS. It turned out that MRS could suppress the inflammation in acetic acid-induced colitis.

Oxidative stress is another prominent factor causing tissue destruction in UC [22]. Reactive oxygen and nitrogen species (ROS and RNS), products of oxidative stress, are the culprits to promote lipid peroxidation, resulting in a negative influence on membrane organization and the structures of proteins and DNA bases [23]. MDA is an end-product from the oxidation of polyunsaturated fatty acids, which is a common marker of oxidative stress besides MPO. On the other hand, SOD and GSH act as inhibitors of oxygen radicals by scavenging oxygen radicals [24]. Thus, we detected the levels of oxidants and antioxidants to assess the degree of oxidative stress in colitis mice. The levels of SOD and GSH were found to be downregulated because of acetic acid, a finding that was consistent with an upregulated level of MDA and MPO. When the colitis mice were treated with MRS, their activities of SOD and GSH were improved and the levels of MDA and MPO were reduced. The result indicated that MRS demonstrated a protective effect when mice showed oxidative stress damage during colitis.

Colonic epithelial cell apoptosis was observed in acetic acid-induced colitis in line with a previous study [25]. Apoptosis has been regarded as a mechanism involving the homeostatic and pathogenic processes of colonic cells in IBD. When apoptosis occurs in epithelial cells, the surrounding histiocytes engulf the programmed death cells and attach to each other to fill the absence. Generally, the local defense is maintained if the quality and quantity of adjacent histiocytes are guaranteed, or inflammation will be activated once uncontrolled apoptosis results in mucosal integrity disruption and bacterial invasion. Additionally, excessive inflammation also leads to the damage of normal histiocytes [26]. It is controversial whether apoptosis or inflammation is the origin for the pathophysiological process of UC. However, apoptosis plays an eminent part in UC, and our TUNEL staining result revealed that MRS was helpful in reducing epithelial cell apoptosis.

To further investigate the mechanism of MRS in colitis, we focused on the TLR4-MyD88 signaling pathway, which is responsible for the expression of inflammatory cytokines and chemokine [27]. When the signals are transferred from TLR4 to MyD88, with the recruitment of IRAK4 and TRAF6 in succession, the IKK complex will be activated, leading to the destruction of I*κ*B by the proteasome. Next, the NF-*κ*B p65 subunit is permitted to translocate into nuclei and promote the production of proinflammatory cytokines such as TNF-*α*, IL-1*β*, and IL-6 [28]. Additionally, the other important TLR4 signaling pathway in charge of the immune response is mediated by MAP kinases such as JNK, ERK, and p38, which are closely correlated with inflammatory mediators such as inducible nitric oxide synthase and cyclooxygenase (COX-2). It was demonstrated that TLR4 recognizes bacterial LPS well and methods to inhibit NF-*κ*B and that MAPK signaling pathways can improve inflammatory damages induced by LPS [29]. The present study showed that acetic acid stimulated the inflammation-related pathway showing upregulated expression levels of TLR4 and MyD88 and subsequent downstream molecules including NF-*κ*B, JNK, ERK, and p38. Moreover, when we treated the colitis mice with MRS, components in the pathway were at lower expression levels. Thus, we speculated that MRS may exert a protective effect in acetic acid-induced colitis via the TLR-4/NF-*κ*B/MAPK signaling pathway.

Moreover, we found that MRS could not only decrease the release of proinflammatory cytokines but also improve the anti-inflammatory ability through the increase in IL-10 production. In fact, several other studies showed that MRS could increase the IL-10 production in different diseases such as postoperative cognitive dysfunction, LPS-induced sepsis, carbon tetrachloride-induced liver injury, and chronic inflammatory pain [30–33]. IL-10/JAK1/STAT3 is a well-documented signaling pathway which can control the anti-inflammatory response. *Lactobacillus rhamnosus* GR-1 supernatant was able to increase IL-10 output and activated the JAK/STAT pathway to exert an anti-inflammatory property in lipopolysaccharide-stimulated placental trophoblast cells [34]. Carboxymethyl chitosan could attenuate inducible nitric oxide synthase and protect against osteoarthritis through the IL-10/JAK1/STAT3 pathway [35]. The expression of IL-10 could inhibit the expression of proinflammatory mediators such as cell surface receptors, chemokines, and cytokines. And the activation of STAT3 could stimulate the expression of anti-inflammatory properties of genes, which in turn suppress the expression of proinflammatory genes [7]. Previous studies showed that MRS may promote upstream PI3K/Akt/GSK-3*β*-mediated IL-10 expression [31, 32]. And our results first revealed that MRS may also promote the downstream IL-10/JAK1/STAT3 signaling pathway to elevate the anti-inflammatory response. In the future, more *in vivo* and *in vitro* studies can be launched to detect and verify the novel effect of MRS on the IL-10/JAK1/STAT3 signaling pathway in different diseases. Because of its simple molecular structure, it is difficult to reveal the detailed mode of action between MRS and the corresponding receptor by traditional experimental methods. However, with the development of the structural biology methods, future work may be focused on the effect of MRS on the structure of influenced proteins such as TLR4, NF-*κ*B, IL-10, JAK, and STAT3.

In summary, we found that methane-rich saline had a protective effect on acetic acid-induced ulcerative colitis, relying on the ability to alleviate oxidative stress and inflammation by inhibiting the TLR4/NF-*κ*B/MAPK signaling pathway and promoting IL-10/JAK1/STAT3-mediated anti-inflammatory response. MRS showed anti-inflammation, antioxidation, and antiapoptosis properties once more in step with previous studies. However, the specific mechanism regarding how methane-rich saline is beneficial remains unclear. It was reported that methane could penetrate the cell membrane and exert its function. Thus, some researchers have predicted that methane acts through proteins embedded in the lipid bilayer of the membrane [36]. Speculation that methane was induced to form methanol or alcohol via a related receptor and then affected the oxidative environment in cells was also proposed to try to explain the biological mechanism. Besides, no appropriate dose of MRS was determined to apply according to previous studies. However, the setting of different doses in our experiment revealed that a high dose at 10 ml/kg performed better than the lower one at 1 ml/kg. However, further research concerning the appropriated dose is required. While ischemia-reperfusion damage was the earliest and most common investigation of methane, diseases like sepsis, hepatitis, and pancreatitis were explored recently. Our research could offer evidence to expand the application area of methane medicine in ulcerative colitis and a novel direction to solve the problem of colitis therapy. There are no certain instructions regarding the physicochemical properties, biological mechanisms, and usage of methane. However, the potential of methane medicine is of great prospect, and further study is warranted.

## Data Availability

The data related to mouse model data, serum cytokine levels, tissue cytokine mRNA levels, oxidative stress indictors, TUNEL staining, and Western blot images used to support the findings of this study are available from the corresponding author upon request.

## References

[1] Iskandar H. N., Dhere T., Farraye F. A. (2015). Ulcerative colitis: update on medical management. *Current Gastroenterology Reports*.

[2] Ungaro R., Mehandru S., Allen P. B., Peyrin-Biroulet L., Colombel J. F. (2017). Ulcerative colitis. *The Lancet*.

[3] Xu B. L., Zhang G. J., Ji Y. B. (2015). Active components alignment of Gegenqinlian decoction protects ulcerative colitis by attenuating inflammatory and oxidative stress. *Journal of Ethnopharmacology*.

[4] Wang Z., Li S., Cao Y. (2016). Oxidative stress and carbonyl lesions in ulcerative colitis and associated colorectal cancer. *Oxidative Medicine and Cellular Longevity*.

[5] Gupta R. A., Motiwala M. N., Mahajan U. N., Sabre S. G. (2018). Protective effect of *Sesbania grandiflora* on acetic acid induced ulcerative colitis in mice by inhibition of TNF-*α* and IL-6. *Journal of Ethnopharmacology*.

[6] Lim S. M., Jeong J. J., Kang G. D., Kim K. A., Choi H. S., Kim D. H. (2015). Timosaponin AIII and its metabolite sarsasapogenin ameliorate colitis in mice by inhibiting NF-*κ*B and MAPK activation and restoring Th17/Treg cell balance. *International Immunopharmacology*.

[7] Hutchins A. P., Diez D., Miranda-Saavedra D. (2013). The IL-10/STAT3-mediated anti-inflammatory response: recent developments and future challenges. *Briefings in Functional Genomics*.

[8] Meng Y., Jiang Z., Li N. (2018). Protective effects of methane-rich saline on renal ischemic-reperfusion injury in a mouse model. *Medical Science Monitor*.

[9] Li Z., Jia Y., Feng Y. (2019). Methane alleviates sepsis-induced injury by inhibiting pyroptosis and apoptosis in vivo and in vitro experiments. *Aging*.

[10] Li Z., Jia Y., Feng Y. (2018). Methane-rich saline protects against sepsis-induced liver damage by regulating the PPAR-*γ*/NF-*κ*B signaling pathway. *Shock*.

[11] Jia Y., Li Z., Feng Y. (2018). Methane-rich saline ameliorates sepsis-induced acute kidney injury through anti-inflammation, antioxidative, and antiapoptosis effects by regulating endoplasmic reticulum stress. *Oxidative Medicine and Cellular Longevity*.

[12] Sun A., Wang W., Ye X. (2017). Protective effects of methane-rich saline on rats with lipopolysaccharide-induced acute lung injury. *Oxidative Medicine and Cellular Longevity*.

[13] Wu J., Wang R., Ye Z. (2015). Protective effects of methane-rich saline on diabetic retinopathy via anti-inflammation in a streptozotocin-induced diabetic rat model. *Biochemical and Biophysical Research Communications*.

[14] Jia Y., Li Z., Liu C., Zhang J. (2018). Methane medicine: a rising star gas with powerful anti-inflammation, antioxidant, and antiapoptosis properties. *Oxidative Medicine and Cellular Longevity*.

[15] de Santana Souza M. T., Teixeira D. F., de Oliveira J. P. (2017). Protective effect of carvacrol on acetic acid-induced colitis. *Biomedicine & Pharmacotherapy*.

[16] Niu X., Zhang H., Li W. (2015). Protective effect of cavidine on acetic acid-induced murine colitis via regulating antioxidant, cytokine profile and NF-*κ*B signal transduction pathways. *Chemico-Biological Interactions*.

[17] Ozbakis Dengiz G., Gursan N. (2005). Effects of *Momordica charantia* L. (*Cucurbitaceae*) on indomethacin-induced ulcer model in rats. *The Turkish Journal of Gastroenterology*.

[18] Guazelli C. F. S., Fattori V., Colombo B. B. (2013). Quercetin-loaded microcapsules ameliorate experimental colitis in mice by anti-inflammatory and antioxidant mechanisms. *Journal of Natural Products*.

[19] Banerjee S., Ghosh S., Sinha K., Chowdhury S., Sil P. C. (2019). Sulphur dioxide ameliorates colitis related pathophysiology and inflammation. *Toxicology*.

[20] Xiao Y. T., Yan W. H., Cao Y., Yan J. K., Cai W. (2016). Neutralization of IL-6 and TNF-*α* ameliorates intestinal permeability in DSS-induced colitis. *Cytokine*.

[21] Mudter J., Neurath M. F. (2007). Il-6 signaling in inflammatory bowel disease: pathophysiological role and clinical relevance. *Inflammatory Bowel Diseases*.

[22] Mitani T., Yoshioka Y., Furuyashiki T., Yamashita Y., Shirai Y., Ashida H. (2017). Enzymatically synthesized glycogen inhibits colitis through decreasing oxidative stress. *Free Radical Biology & Medicine*.

[23] Kruidenier L., Kuiper I., van Duijn W. (2003). Imbalanced secondary mucosal antioxidant response in inflammatory bowel disease. *The Journal of Pathology*.

[24] Margaritelis N. V., Veskoukis A. S., Paschalis V. (2015). Blood reflects tissue oxidative stress: a systematic review. *Biomarkers*.

[25] Zeissig S., Bojarski C., Buergel N. (2004). Downregulation of epithelial apoptosis and barrier repair in active Crohn’s disease by tumour necrosis factor alpha antibody treatment. *Gut*.

[26] Qiu W., Wu B., Wang X. (2011). PUMA-mediated intestinal epithelial apoptosis contributes to ulcerative colitis in humans and mice. *The Journal of Clinical Investigation*.

[27] Kawai T., Akira S. (2010). The role of pattern-recognition receptors in innate immunity: update on Toll-like receptors. *Nature Immunology*.

[28] Tsubaki M., Takeda T., Kino T. (2015). Mangiferin suppresses CIA by suppressing the expression of TNF-*α*, IL-6, IL-1*β*, and RANKL through inhibiting the activation of NF-*κ*B and ERK1/2. *American Journal of Translational Research*.

[29] Gao Y., Jiang W., Dong C. (2012). Anti-inflammatory effects of sophocarpine in LPS-induced RAW 264.7 cells via NF-*κ*B and MAPKs signaling pathways. *Toxicology In Vitro*.

[30] Zhang D., Li N., Wang Y. (2019). Methane ameliorates post-operative cognitive dysfunction by inhibiting microglia NF-*κ*B/MAPKs pathway and promoting IL-10 expression in aged mice. *International Immunopharmacology*.

[31] Zhang X., Li N., Shao H. (2016). Methane limit LPS-induced NF-*κ*B/MAPKs signal in macrophages and suppress immune response in mice by enhancing PI3K/AKT/GSK-3*β*-mediated IL-10 expression. *Scientific Reports*.

[32] Yao Y., Wang L., Jin P. (2017). Methane alleviates carbon tetrachloride induced liver injury in mice: anti-inflammatory action demonstrated by increased PI3K/Akt/GSK-3*β*-mediated IL-10 expression. *Journal of Molecular Histology*.

[33] Zhou S. Z., Zhou Y. L., Ji F. (2018). Analgesic effect of methane rich saline in a rat model of chronic inflammatory pain. *Neurochemical Research*.

[34] Yeganegi M., Leung C. G., Martins A. (2010). Lactobacillus rhamnosus GR-1-induced IL-10 production in human placental trophoblast cells involves activation of JAK/STAT and MAPK pathways. *Reproductive Sciences*.

[35] Kong Y., Zhang Y., Zhao X., Wang G., Liu Q. (2017). Carboxymethyl-chitosan attenuates inducible nitric oxide synthase and promotes interleukin-10 production in rat chondrocytes. *Experimental and Therapeutic Medicine*.

[36] Fink M. P. (2012). Pharmacological effects of inhaled methane: plausible or not?. *Critical Care Medicine*.

